# Phantosmia, Parosmia, and Dysgeusia Are Prolonged and Late-Onset Symptoms of COVID-19

**DOI:** 10.3390/jcm10225266

**Published:** 2021-11-12

**Authors:** Sophia E. Schambeck, Claudia S. Crowell, Karolin I. Wagner, Elvira D’Ippolito, Teresa Burrell, Hrvoje Mijočević, Ulrike Protzer, Dirk H. Busch, Markus Gerhard, Holger Poppert, Henriette Beyer

**Affiliations:** 1 Helios Klinikum München West, Steinerweg 5, 81241 München, Germany; holger.poppert@helios-gesundheit.de (H.P.); Beyer.henriette@googlemail.com (H.B.); 2 Institute for Medical Microbiology, Immunology and Hygiene, Technical University of Munich, Trogerstr. 30, 81675 München, Germany; claudia.crowell@tum.de (C.S.C.); ki.wagner@tum.de (K.I.W.); elvira.dippolito@tum.de (E.D.); teresa.burrell@tum.de (T.B.); dirk.busch@tum.de (D.H.B.); markus.gerhard@tum.de (M.G.); 3 German Center for Infection Research (DZIF), Partner Site Munich, 81675 Munich, Germany; protzer@tum.de; 4 Institute of Virology, School of Medicine, Technical University of Munich, Trogerstr. 30, 81675 München, Germany; hrvoje.mijocevic@tum.de; 5 Klinik und Poliklinik für Neurologie im Neuro-Kopf-Zentrum, Klinikum Rechts der Isar, Ismaninger Str. 22, 81675 München, Germany

**Keywords:** parosmia, phantosmia, dysgeusia, smell, taste, coronavirus, COVID-19, long-COVID

## Abstract

Deficiencies in smell and taste are common symptoms of COVID-19. Quantitative losses are well surveyed. This study focuses on qualitative changes such as phantosmia (hallucination of smell), parosmia (alteration of smell), and dysgeusia (alteration of taste) and possible connections with the adaptive immune system. Subjective experience of deficiency in taste and smell was assessed by two different questionnaires after a median of 100 and 244 days after first positive RT-PCR test. SARS-CoV-2-specific antibody levels were measured with the iFlash-SARS-CoV-2 assay. After 100 days a psychophysical screening test for olfactory and gustatory dysfunction was administered. 30 of 44 (68.2%) participants reported a chemosensory dysfunction (14 quantitative, 6 qualitative, 10 quantitative, and qualitative) during COVID-19, eleven (25.0%) participants (1 quantitative, 7 qualitative, 3 quantitative, and quantity) after 100 days, and 14 (31.8%) participants (1 quantitative, 10 qualitative, 3 quantitative and qualitative) after 244 days. Four (9.1%) participants, who were symptom-free after 100 days reported now recently arisen qualitative changes. Serological and T-cell analysis showed no correlation with impairment of taste and smell. In conclusion, qualitative changes can persist for several months and occur as late-onset symptoms months after full recovery from COVID-19-induced quantitative losses in taste and smell.

## 1. Introduction

Early in the coronavirus-disease 2019 (COVID-19) pandemic several studies showed that deficiencies in smell and taste are frequent symptoms in patients diagnosed with severe acute respiratory syndrome coronavirus-2 (SARS-CoV-2) infection and these symptoms can be used as early markers of the disease [1,2,3]. In contrast to loss of smell in the context of a common cold, olfactory dysfunction in COVID-19 is rarely associated with nasal congestion [4,5]. The prevalence of smell and taste dysfunction ranges between 18.6 and 90% in SARS-CoV-2 positive patients in Europe and America [6,7]. Quantitative losses in smell (hyposmia and anosmia) and taste (ageusia) have been well described [8,9]. To date there are case reports on qualitative changes in olfactory and gustatory function, such as phantosmia (hallucination of smell), parosmia (alteration of smell), and dysgeusia (alteration of taste) [10,11]. Few publications systematically study the long-term persistence of qualitative change [12] and most of these focus on parosmia [13,14]. Patients with persisting olfactory dysfunction report a lower quality of life due to depression, anxiety, interference with daily routines and problems with enjoyment of food [15,16,17]. Qualitative dysfunctions seem to have a higher impact on quality of life than quantitative dysfunctions [17] and should therefore not be disregarded in clinical practice. Our substudy focuses on qualitative changes in olfactory and gustatory function after infection with SARS-CoV-2. Here, we determined the prevalence and nature of olfactory and gustatory dysfunction during COVID-19 illness, as well as after a median of 100 and 244 days. Furthermore, we provide detailed descriptions of the participants’ subjective experience of phantosmia, parosmia, and dysgeusia. Antibody levels were tracked over the course of the study to investigate possible correlations of immune response and impairments of taste and smell.

## 2. Materials and Methods

### 2.1. Participants

In total, data were obtained from 44 subjects. The cohort included 40 hospital employees who acquired SARS-CoV-2 infection at Helios Klinikum München West in Germany. In addition, four relatives of these health-workers infected by their partners were included. Participation was voluntarily and not remunerated.

### 2.2. EPI-SARS Study and the Substudy on Smell and Taste

**Figure 1:**
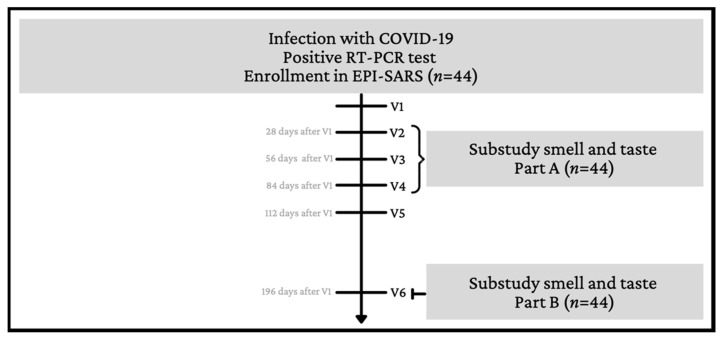
Flow chart showing the structure and links between the parental study (EPI-SARS study) and the substudy on smell and taste. RT-PCR positive tested individuals were included in the study and followed over the course of 6 months, with repeat blood sampling and questionnaires (V1–V6). The smell and taste substudy was part of the EPI-SARS study protocol. Part A, including Q1 and psychophysical screening test, and part B with Q2 were conducted at V2–4 and V6 respectively.

This substudy about smell and taste deficiency was conducted within the framework of the study “Establishment and validation of epitope-specific SARS-CoV-2 blood-based testing methods” (EPI-SARS). For EPI-SARS, whole blood and serum samples were taken and questionnaires on demographic information and interim RT-PCR test results administered at six study visits (V1–V6, see Figure 1) between May and December 2020.

The substudy about smell and taste started in July 2020, and comprised all employees recruited at our unit, the Helios Klinikum Munich. Timepoints were defined by the parental study EPI-SARS. Part A of the substudy, including psychophysical Sniffin’ sticks screening and Taste stripes test, as well as questionnaire Q1 was conducted either during V2, V3, or V4 of EPI-SARS. Part B of the substudy, comprising questionnaire Q2 was conducted during V6. Varying intervals between SARS-CoV-2 infection and enrollment in the parental study caused the participants’ broad range of days.

Inclusion criteria for EPI-SARS and the substudy were a positive RT-PCR test for SARS-CoV-2 and age ≥18 years. Olfactory or gustatory dysfunctions in a patient that were known prior to the onset of the pandemic were an exclusion criteria for the substudy.

### 2.3. Categories of Disease Severity

Severity of illness was divided into four categories. The category “asymptomatic”, included participants who reported no symptoms or only tiredness without any other symptoms. “Mild” included patients with symptoms such as a cold, cough, diarrhea, and others without fever. The third category included patients who had a fever (≥38 °C) but were not hospitalized. The last category comprised patients who were hospitalized.

### 2.4. Evaluation of Antibody Levels

SARS-CoV-2 specific IgG antibody levels were evaluated over the course of the study (V1-V6) using the iFlash-SARS-CoV-2 IgG chemiluminescence immunoassay kits (Shenzhen YHLO Biotech Co., Ltd. (Shenzhen, China)). The assays were performed on the iFLASH immunoassay analyzer (Shenzhen YHLO Biotech Co., Ltd. (Shenzhen, China)) using the manufacturer’s protocol. Spike protein and nucleocapsid protein were detected. The assay procedure was described by Qian C et al. [18]. The cutoff value was >10 AU/mL.

### 2.5. T Cell Analysis

For T-cell analysis, peripheral blood mononuclear cells (PBMCs) were isolated from whole blood by density centrifugation. T cells were expanded for 12 days on a peptide pool stimulation. The in-house designed peptide pool contains diverse epitopes predicted for the most common HLA class I molecules across various domains of the SARS-CoV-2 proteome. T-cell immunity was investigated via intracellular cytokine release in peptide stimulation assays. The assay is described elsewhere [19].

### 2.6. Questionnaires Q1 and Q2

The structured questionnaires Q1 and Q2 for measuring the subjective experience of taste and smell were self-developed, since COVID-19 was an unexplored disease and we wanted to give room for detailed descriptions and yet unknown symptoms. Q1 and Q2 both addressed chemosensory function retrospectively during COVID-19. Intra-participant results could therefore be compared. Inconsistent replies were followed up.

During part A of the substudy Q1 measured self-reported smell and taste function 100 days after infection as well as retrospectively during acute COVID-19 infection. Participants were asked if they had experienced a dysfunction in taste or smell at the beginning of the illness and how long it lasted; they had three options to choose from: “<7 days”, “>7 days” or “other”. Changes were assessed by the questions “Are there specific smells you sensed enhanced or diminished?” and “Are or were specific tastes more severely affected?” (“no”, or if “yes” was selected, participants would further specify with the options “sweet/sour/salty/bitter”). Participants were asked if they still experienced any changes in taste and if “yes” since when. The same question was applied to smell. The question “Did you notice any other changes in smell or taste” (“no”, or if “yes, please describe”) was formulated as an open-ended question to give space for unexpected symptoms and detailed subjective descriptions.

Q1 revealed that several participants suffered from qualitative changes in smell and taste after the infection. To confirm this hypothesis and specifically address this finding, part B of the substudy with the second questionnaire (Q2) was developed.

In Q2 subjects were again asked if they had experienced a dysfunction in smell during the illness. Changes in quality of smell were assessed by the questions “Were certain smells changed?” (“no”, or if “yes” was selected participants would describe which smells were changed and in which way). Information about phantosmia during acute COVID-19 infection was gathered with the question “Did you smell anything that wasn’t there? (Did you hallucinate smell(s)?)”. In the case of such sensations participants were asked to name the hallucinated smell(s) and describe their experience of phantosmia. The answer options were “pleasant“ or “unpleasant“ as well as “known“ or “unknown“. Gustatory function during COVID-19 was assessed by the question if participants had experienced a dysfunction in taste during the acute illness (“no”, or if “yes” was selected participants would further specify with the options “diminished”/“changed”/“hallucinated” and in addition describe the sensation.). The final question was how participants currently evaluated their olfactory and gustatory function. The answer options for taste as well as for smell were: “as before”, “changed”, “hallucinatory” or “diminished”. Furthermore, information about previous otorhinological illnesses (surgery, allergic rhinitis, …) and its impact on the sense of smell, as well as prior smell dysfunctions in the context of viral infections was collected.

### 2.7. Sniffin’ Sticks Screening 12 Test with Taste Stripes

Objective information was collected with the Sniffin’ Sticks Screening 12 test with taste stripes [20]. In the Sniffin’ Sticks Screening 12 test odor identification is assessed for 12 common smells. It is widely used for rapid screening of olfactory dysfunction in clinical practice and normative tests have been performed in several countries [21]. Identification of individual odors was performed from multiple-choice lists of four, after the “forced-choice technique”, whereby the subjects had to decide for one odor even in cases of uncertainty. Scores range from 0 to 12.

To assess taste, tasting stripes with the four main taste qualities sweet, sour, bitter and salty were placed on specific tongue areas. Participants cleared their mouth of the previous taste with a sip of water before each new strip. Afterwards, they noted down how they perceived the quality.

During the taste and smell assessment participants did not receive any feedback on their answer. A test result was considered abnormal if at least one of the four qualities was not identified correctly. At the time of testing all participants were fully recovered from their COVID-19 infection and had already received two negative RT-PCR test results.

### 2.8. Statistical Analysis

Statistical analysis was performed using R^®^ (The R foundation for Statistical Computing, Version 4.0.3). Categorical data are shown in absolute and relative frequencies, with the respective maxima and minima, their standard deviation (SD), and the distribution of values. The Wilcoxon test was applied for intergroup comparisons of antibody titers and T-cell response between participants with and participants without alteration of taste and smell (statistical significance set at *p* < 0.05).

## 3. Results

### 3.1. Cohort Characteristics

All 44 participants tested positive by COVID-19 RT-PCR. Time of most infections was early April 2020 (median 1 April 2020, earliest 23 March 2020, latest 10 June 2020).

Part A of the substudy, including Q1 and psychophysical screening tests was conducted after a median (range: min–max) of 100 (55–132) days; part B with Q2 after a median of 244 (180–279) days.

For the characteristics of the cohort see Table 1.

### 3.2. Self-Reported Alteration of Taste and Smell over the Course of the Study

#### 3.2.1. Overview of Alteration of Taste or Smell

Overall, 30 of 44 (68.2%) participants reported an alteration of taste or smell (14 quantitative, 6 qualitative, 10 quantitative and qualitative) during their acute COVID-19 illness. For the constellation of reported symptoms see Table A1. Of the ten participants with a combined qualitative and quantitative dysfunction three reported this combined dysfunction from symptom onset. The other seven participants experienced a quantitative loss for several days, before their sense of smell or taste returned qualitatively changed. In 8 of the 30 (26.7%) cases with alterations of taste or smell, the disorder ceased within seven days.

**Figure 2:**
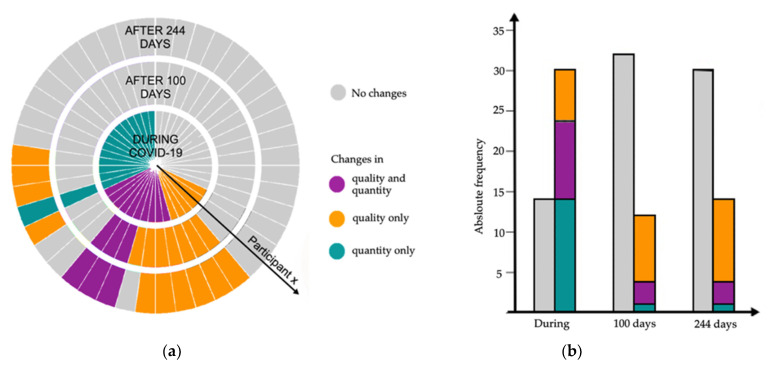
Quantitative and qualitative alterations in taste and smell of all 44 participants over the course of the study. Data on chemosensory dysfunction during the disease were collected in retrospectively, whereas information concerning 100 and 244 days were collected prospectively. (**a**) Development of quantitative and qualitative alterations of taste and smell of each participant. Each slice symbolizes one participant and the respective alteration of taste and smell during COVID-19 infection, as well as 100 and 244 days later. (**b**) Absolute frequencies and distribution of alterations in taste and smell over the course of study.

After a median (range: min–max) of 100 (55–132) days, eleven (25.0%) participants reported an ongoing alteration of these senses (1 quantitative, 7 qualitative, 3 quantitative and qualitative). Figure 2a shows each participants’ development of qualitative and quantitative alterations of smell and taste over the course of the study.

After a median of 244 (180–279) days, 14 (31.8%) participants (1 quantitative, 10 qualitative, 3 quantitative and qualitative) reported alterations of taste or smell. For ten (22.7%) participants, the disorder has been persisting since the acute COVID-19 illness (for a median of 250 (229–255) days). Four (18.2%) participants, who had suffered from quantitative losses (3 quantitative, 0 qualitative, 1 quantitative, and qualitative) during the acute illness and had been fully recovered by the study visit after 100 days, reported now newly occurring qualitative alterations of taste and smell. None of these four participants was reinfected with SARS-CoV-2, nor did any of these subsequently test positive again by RT-PCR test, which was conducted regularly within the scope of hospital screenings. 

The frequencies of qualitative and quantitative alterations over the course of study are visible in Figure 2b. Table A1 shows the reported frequencies of qualitative changes (phantosmia, parosmia, and dysgeusia), quantitative changes (anosmia, hyposmia, ageusia, hypogeusia), and constellations of symptoms during COVID-19 as well as after a median of 100 and 244 days.

#### 3.2.2. Description of Qualitative Changes with Delayed Onset

After a median of 244 (180–279) days four (9.1%) participants reported newly occurring qualitative changes. These participants had suffered from chemosensory dysfunction during COVID-19 (3 quantitative, 0 qualitative, 1 quantitative, and qualitative) and had been fully recovered after several days, as well as at their study visit after a median of 100 days. Now they reported phantosmia (1), phantosmia and dysgeusia (1), parosmia and dysgeusia (1) as well as dysgeusia (1). Furthermore, one participant who had suffered from dysgeusia since the acute COVID-19 illness described a recently arisen additional parosmia. 

#### 3.2.3. Subjective Alteration of Taste Qualities during COVID-19

Concerning taste, certain qualities were more severely affected in twelve cases (27.3%) (of these nine with reported dysgeusia, three with reported hypogeusia). A combined alteration of more qualities occurred with 6 reported cases as often as the alteration of only one quality. 

#### 3.2.4. Subjective Descriptions of Phantosmia, Parosmia and Dysgeusia

Over the course of the study, nine (20.5%) different participants experienced phantosmia. Eight experienced the hallucinated smells as unpleasant. Four of these eight participants described the hallucinated smells as known and nameable (cigarette smoke (1), fire smoke & feces (1), feces & foul (1), sauerkraut (1)).

The eight (18.2%) participants with parosomia experienced a changed smell of perfume (3), feces & perfume (1), disinfectant (1), detergent (1), and bread & coffee (1). In these seven cases the parosmia was perceived as negative and nauseating. For one participant everything smelled like roasted chicken (1) without negative effect on his well-being.

Of the eleven (25.0%) participants experiencing dysgeusia, five mentioned that they lost the ability to differentiate tastes in mixed dishes. Furthermore, a bitter aftertaste (1), a bitter (1), salty (1) or saltless (2) taste of food, a burning sensation while eating (1) and a change in sweet dishes (3) were described. Food and drink preferences changed in four cases.

### 3.3. Psychophsycial Screening Tests after a Median of 100 Days after Positive RT-PCR Test

#### 3.3.1. Odor Identification Screening Test

**Figure 3:**
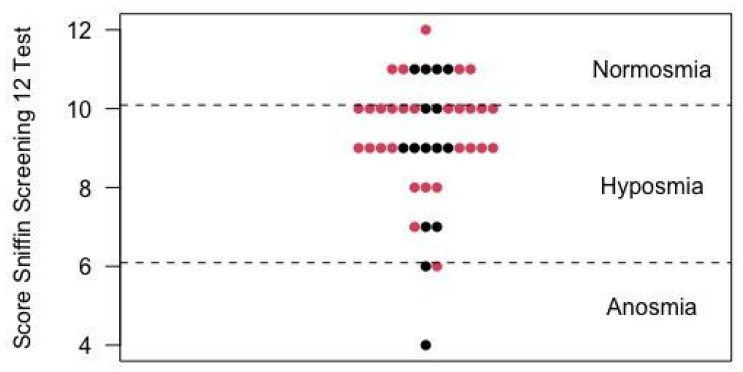
Scores odor identification test of the 44 participants (red: women, black: men).

Normosmia (score of 11 or 12) was present in nine (20.5%) participants. Thirty-two (72.7%) cases had a score within the range of hyposmia (score of 7 to 10). Anosmia (score of 0 to 6) occurred in three (6.8%) cases. Figure 3 shows the distribution of the scores of all participants. None of the nine (20.5%) participants who described an ongoing subjective alteration of smell in Q1 reached normosmia. Six (13.6%) were within the range of hyposmia, the other three (6.8%) were anosmic.

#### 3.3.2. Taste Identification Screening Test

In total, 26 of 44 (59.1%) participants showed a normal test result, whereas 18 (40.9%) participants were not able to identify all four taste qualities correctly. In ten (22.7%) cases only one specific quality was not recognized, whereas eight (18.1%) participants failed to identify more than one quality correctly. The quality “salt” was not recognized eleven times, “sour” eight times, “bitter” six times, and “sweet” three times. Even though only six (13.6%) participants reported an ongoing subjective alteration of taste in Q1, 18 (40.9%) showed an abnormal test result. Of the six (13.6%) participants reporting an ongoing dysgeusia in Q1, five (11.4%) showed an abnormal test result.

### 3.4. Immune Response in Context with Alteration of Taste and Smell

**Figure A1:**
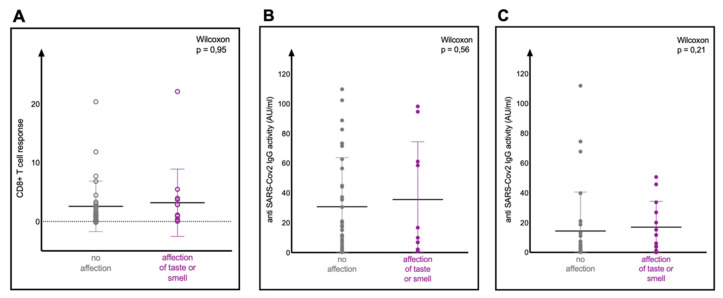
(**A**) CD8+ T-cell response (mean 63 days after positive RT-PCR test) of the participants with or without alteration of taste and smell ongoing after a median of 244 days after positive RT-PCR test. (**B**,**C**) Anti SARS-Cov2 IgG levels of the participants with or without alteration of taste and smell ongoing at 100 days (A1B) and 244 days (A1C) after positive RT-PCR test. Data analysis with the Wilcoxon test showed no significant difference between both groups.

A comparison of SARS-CoV-2 specific IgG antibody reactivity measured at the time of part A and part B of the substudy showed no correlation with the alteration of taste or smell. Most participants also showed SARS-CoV-2 specific CD8+ T-cell responses upon peptide stimulation, but no correlation with the symptoms of taste or smell was evident (see Figure A1A–C in Appendix A).

## 4. Discussion

This study adds to the existing literature on COVID-19-associated alteration of taste and smell by investigating quantitative and qualitative disorders not only in the acute phase of infection but also after a median of 100 and 244 days after a positive RT-PCR test. Focusing on the nature of qualitative changes in taste and smell in our second questionnaire (Q2), this study reports on the subjective experience of phantosmia, parosmia, and dysgeusia after infection with SARS-CoV-2. Our study is unique in its detailed descriptions of the subjective experience of phantosmia, parosmia, and dysgeusia in the context of COVID-19. Antibody titers were measured alongside olfactory and gustatory function to study possible connections between the adaptive immune system and SARS-CoV-2 induced taste and smell dysfunctions.

Consistent with several other studies, our study corroborates that dysfunctions of taste and smell are early onset symptoms of COVID-19 [22,23,24,25] and persist in many cases for several months [13,26,27,28]. It was evident that qualitative changes (parosmia, phantosmia and dysgeusia) can occur as late-onset symptoms. In four cases, parosmia and phantosmia arose after months of complete absence of other symptoms. In addition, the data suggest that these qualitative changes are probably not as rare as assumed at earlier stages of the pandemic [29]. It is striking that whereas the largest part of this study’s participants recovered from their isolated quantitative changes such as anosmia, hyposmia, ageusia and hypogeusia within a few weeks after the illness, most participants suffered from qualitative changes for several months. Overall, 29.5% (13 of 44 (see Figure 2 and Table A1)) of our cohort reported qualitative changes after 244 days. This high occurrence indicates that qualitative changes are common long-term symptoms in COVID-19 patients. Another study systematically investigating qualitative changes reported qualitative alterations of smell and/or taste in 6 of 17 (35.3%) COVID-19 patients after 99 ± 30.1 days after negative nasopharyngeal swab [12]. The validity of these numbers is compromised by the small cohorts (*n* = 44 of our study and *n* = 17 of Ercoli et al. [12]). Yet, taking smell as an example, those prevalences are not unrealistically high, since in general about one-third of patients with impairment of olfactory function of various causes also show parosmia and phantosmia [30,31]. Hopkins et al. measured a prevalence of parosmia of 43.1% (among those with a self-reported loss of smell at the onset of the disease) after six months [13], whereas this study’s prevalence of parosmia was 25% (6 cases of parosmia of 24 participants with self-reported smell dysfunction at the onset of disease (see Appendix A Table A1) after 244 days. Qualitative alterations of the gustatory and olfactory senses usually become evident several days to months after the acute illness and quantitative changes [13,32]. Therefore, qualitative changes could probably not be fully captured in studies assessing smell and taste dysfunctions only during or shortly after the acute stage of COVID-19 illness, resulting in the reports of low prevalence of these symptoms [29].

Information on chemosensory function during COVID-19 was collected in retrospect since the questionnaires on taste and smell were only applied after 100 (Q1) and 244 (Q2) days. This might have made the assessment of prevalence of chemosensory dysfunctions during COVID-19 infection inaccurate. To lessen the impact of the lack of baseline measurement, each participant was asked to describe their chemosensory function during the acute illness in both questionnaires. Inconsistent replies were followed up.

A detailed comparison and quantification of the constellations of symptoms in patients between timepoints 100 and 244 is precluded by the usage of two different questionnaires (Q1 and Q2). Whereas Q2 assessed qualitative changes systematically, Q1 assessed if patients still suffered from any changes in taste or smell and asked to describe any noticed changes in taste or smell. From the answers and subjective descriptions of their alterations, we could deduce if participants suffered from quantitative or qualitative or combined changes in taste or smell. Even though inconsistent instruments were used over time the collected information allows the conclusion that qualitative changes can occur as late-onset symptoms. Participants concerned reported quantitative losses during COVID-19, a full recovery after a median of 100 days, and then described recently developed hallucinations and alterations in smell or taste in Q2.

After a median of 100 days a psychophysical screening test of olfactory and gustatory function was applied to objectively assess the participants’ self-reported ability to smell and taste. For this, the Sniffin’ Screening12 and Taste Stripes tests, which have been validated in numerous studies and vastly used in clinical practice, were chosen [21,33]. As screening tools these tests allow for the detection of abnormalities in smell and taste function but do not provide a detailed assessment of chemosensory function. 100 days after RT-PCR positivity, self-reported subjective alterations of taste or smell were confirmed by abnormal objective test results. Both in the Sniffin’ Screening12 test and the Taste Stripes test, a larger proportion of participants showed an objective alteration than reported in the questionnaires. Studies have shown a higher prevalence of olfactory and gustatory dysfunction in patients using psychophysical tests compared with self-reports [25,27]. This phenomenon maybe partly due to the revelation of preexisting smell and taste disorders unrelated to COVID-19 [34]. Nevertheless, the large discrepancy (smell dysfunction after a median of 100 days: 32 (72.7%) psychophysical test vs. 8 (18.2%) self-reported) suggests that many patients are not aware or have already adapted to their by COVID-19 altered sense of smell. This stresses the necessity of psychophysical tests in clinical practice to properly assess chemosensory function.

The investigation of antibody titers and T-cell response after a median of 100 and 244 days showed no correlation with impairment of smell or taste (see Figure A1A–C). This indicates that the mechanism of COVID-19 caused disorders of taste and smell is not directly a result of either inactivity or overactivity of these arms of the adaptive immune system but caused by a more localized process. Studies propose that the infection of supporting cells in the mucosa and damage of vascular pericytes cause a localized inflammation and cytokine release resulting in neuronal dysfunction [35,36]. However, the persistence of olfactory loss together with the occurrence of qualitative symptoms such as parosmia [13,27] suggests the possibility of direct damage of olfactory receptor cells or central pathways. The involvement of central structures is also indicated by a neuroimaging study revealing hypometabolism in intracranial olfactory structures in patients with parosmia after infection with SARS-CoV-2 [37]. The literature on direct neurotropism of SARS-CoV-2 is conflicting. Coronavirus antigen and SARS-CoV-2 RNA have been found in olfactory neurons and brains of postmortem COVID-19 patients [38], however, a recent review by Butowt et al. concluded that direct infection of neuronal cells by SARS-CoV-2 is unlikely [39]. Hence, evidence for direct neurotropism of SARS-CoV-2 and its possible route to the brain need further investigation.

**Figure A2:**
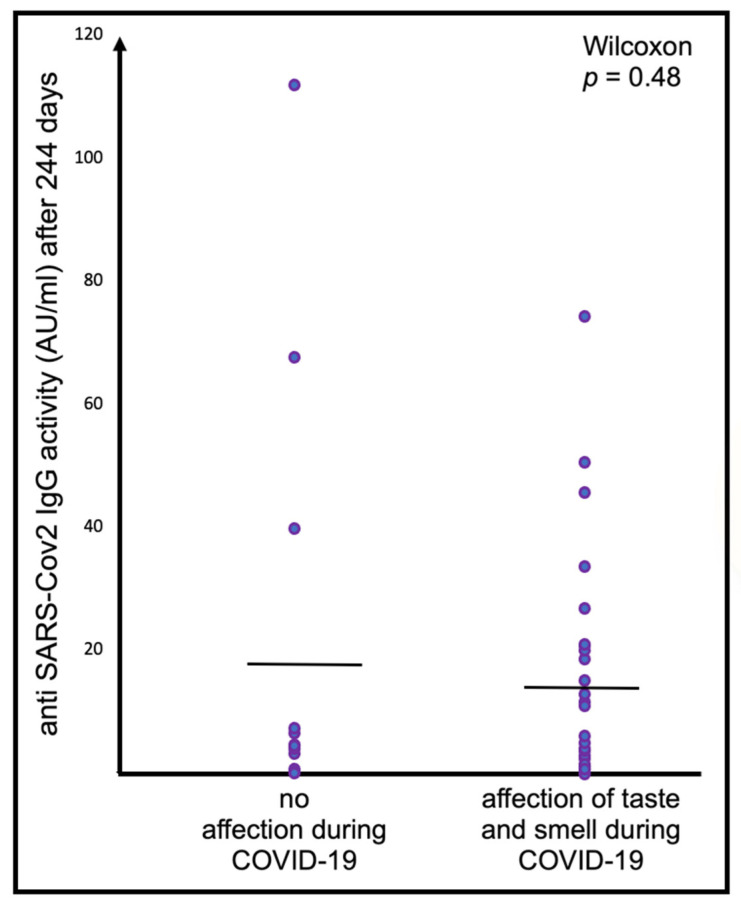
Anti SARS-Cov2 IgG levels after a median of 244 days after positive RT-PCR test of the participants with or without alteration of taste and smell during the illness. Data analysis with the Wilcoxon test showed no significant difference between both groups.

Data on the connection between antibody titer and symptoms of smell and taste are also conflicting. A study by Dehgani-Mobaraki et al. tracked antibody levels (against S-RBD) of 32 recovered COVID-19 patient. Subjects who reported smell and taste loss during SARS-CoV-2 infection, with respect to disease severity developed significantly higher antibody titers at 14 months (*p* = 0.043 and *p* = 0.031 respectively) [40]. In contrast, we found no significant difference in antibody levels of patients with and without reported smell and taste loss during the disease and after a median of 244 days (see Figure A2). Investigations with larger participant numbers are needed.

Patients with persisting olfactory dysfunction of various causes report a lower quality of life due to depression, anxiety, disruption of daily routines, and problems with enjoyment of food [15,16,17,41]. Qualitative dysfunctions seem to have a stronger negative impact on life quality than quantitative dysfunctions [17]. To improve and regain function of smell, olfactory training can be applied. Evidence shows that this treatment option significantly improves olfactory function after postinfectious dysfunction [42,43,44,45]. Furthermore, persistent olfactory impairment after COVID-19 might be a marker for an increased long-term risk of neurodegenerative disorders [46]. It is important to pay attention to these symptoms and its possible impact on the patients’ well-being in clinical practice and keep treatment options in mind.

### Limitations

The cohort of 44 participants is small and the healthy worker effect is applicable since all participants are employees and able to perform their healthcare duties. However, one of its strengths is that all of the participants were recruited from the same location and had experienced COVID-19 infection at a similar point in time.

The questionnaires were self-developed and the usage of two different questionnaires after the median of 100 (Q1) and 244 (Q2) days precludes detailed comparisons of the constellations of symptoms between these two timepoints. In addition, these two timepoints correspond to a broader time range, which influences the comparability between participants and the reliability of the results. The singular occurrence of many constellations of symptoms (see Table A1) comprises the interpretation of the study as well.

## 5. Conclusions

In conclusion, COVID-19-induced phantosmia, parosmia, and dysgeusia can persist for longer periods. Furthermore, these qualitative alterations in smell and/or taste can occur with a latency of several months after full recovery from COVID-19-caused quantitative losses of smell and taste. More prospective studies with larger cohorts are needed to study these phenomena.

## Figures and Tables

**Table 1:** Demographic and anamnestic information about the cohort of the substudy.

Parameter	Number of Participants (%)
Sample size	44 (100%)
Gender	29 F (65.9%) 15 M (34.1%)
Age at part A of the substudy (median, range)	41, 23–62
Active smoker	5 (11.3%)
Severity of COVID-19 illness	Asymptomatic 6 (13.6%) Mild 24 (54.5%) With Fever 13 (29.5%) Hospitalized 1 (2.3%)

## Data Availability

The authors declare that the data supporting the findings of this study are available within the article and appendix as well as from the corresponding author upon reasonable request. Source data for Figure A1A–C and Figure A2 will be published elsewhere; the link will be provided upon publication.

## Appendix A

**Table A1:** Table A1 shows the reported frequencies of qualitative changes (phantosmia, parosmia, and dysgeusia), quantitative changes (anosmia, hyposmia, ageusia, hypogeusia), and which sense was affected during COVID-19 as well as after a median of 100 and 244 days. Data concerning smell and taste during COVID-19 were collected in retrospective. Data concerning timepoint “100 days” was collected by questionnaire Q1, data concerning “244 days” was collected by questionnaire Q2. The two categories “Asymptomatic” and “Alteration of taste or smell” (dark grey background) contain together all 44 study participants. The category “Alteration of taste or smell” is split up in the three groups “Quantity only”, “Quality only” and “Quantity and quality” (light grey background). Each of these three groups is subdivided in “Taste only”, “Smell only” and “Taste and smell” with the respective symptom constellations (in italics).

Description	During COVID-19	100 Days	244 Days
Asymptomatic	14	33	30
Alteration of taste or smell	30	11	14
Quantity only (total)	14	1	1
Taste only	1	1	0
*Ageusia (ag.)*	*1*	*0*	*0*
*Hypogeusia (hg.)*	*0*	*1*	*0*
Smell only	0	0	0
Taste and smell	13	0	1
*Ag. and anosmia (an.)*	*13*	*0*	*0*
*Hg. and hyposmia (Ho.)*	*0*	*0*	*1*
Quality only (total)	6	7	10
Taste only—Dysgeusia (dy.)	5	2	2
Smell only	0	4	3
*Parosmia (pa.)*	*0*	*2*	*1*
*Phantosmia (ph.)*	*0*	*1*	*2*
*Pa. and ph.*	*0*	*1*	*0*
Taste and smell	1	1	5
*Dy., pa. and ph.*	*1*	*1*	*2*
*Dy. and pa.*	*0*	*0*	*2*
*Dy. and ph.*	*0*	*0*	*1*
Quality and quantity (total)	10	3	3
Taste only	0	0	0
Smell only—pa. and ho.	0	1	1
Taste and smell	10	2	2
*Dy. and an.*	*3*	*0*	*0*
*Dy., pa. and ph.*	*1*	*0*	*0*
*Dy., an., pa. and ph.*	*1*	*0*	*0*
*Ag., an. and pa.*	*1*	*0*	*0*
*Ag., an. and ph.*	*1*	*0*	*0*
*Ag., an., pa. and ph.*	*3*	*0*	*0*
*Dy., hg. and ho.*	*0*	*1*	*1*
*Dy., ho., pa. and ph.*	*0*	*1*	*0*
*Dy. and ho.*	*0*	*0*	*1*

## References

[1] Lee Y., Min P., Lee S., Kim S.-W. (2020). Prevalence and Duration of Acute Loss of Smell or Taste in COVID-19 Patients. J. Korean Med. Sci..

[2] Reinhard A., Ikonomidis C., Broome M., Gorostidi F. (2020). Anosmia and COVID-19. Rev. Med. Suisse.

[3] Kain P. (2020). Loss of Smell and Taste: Potential of Using Them as Markers for Early Detection of COVID-19. Adv. Neurol. Neurosci..

[4] Huart C., Philpott C., Konstantinidis I., Altundag A., Whitcroft K., Trecca E., Cassano M., Rombaux P., Hummel T. (2020). Comparison of COVID-19 and common cold chemosensory dysfunction. Rhinology.

[5] Glezer I., Bruni-Cardoso A., Schechtman D., Malnic B. (2021). Viral infection and smell loss: The case of COVID-19. J. Neurochem..

[6] Rojas-Lechuga M.J., Izquierdo-Domínguez A., Chiesa-Estomba C., Calvo-Henríquez C., Villarreal I.M., Cuesta-Chasco G., Bernal-Sprekelsen M., Mullol J., Alobid I. (2021). Chemosensory dysfunction in COVID-19 out-patients. Eur. Arch. Otorhinolaryngol..

[7] Santos R.E.A., da Silva M.G., Barbosa D.A.M., Gomes A.L.D.V., Galindo L.C.M., Aragão R.D.S., Ferraz-Pereira K.N. (2021). Onset and duration of symptoms of loss of smell/taste in patients with COVID-19: A systematic review. Am. J. Otolaryngol..

[8] Horvath L., Lim J.W.J., Taylor J.W., Saief T., Stuart R., Rimmer J., Michael P. (2021). Smell and taste loss in COVID-19 patients: Assessment outcomes in a Victorian population. Acta Otolaryngol..

[9] D’Ascanio L., Pandolfini M., Cingolani C., Latini G., Gradoni P., Capalbo M., Frausini G., Maranzano M., Brenner M.J., Di Stadio A. (2021). Olfactory Dysfunction in COVID-19 Patients: Prevalence and Prognosis for Recovering Sense of Smell. Otolaryngol. Head Neck Surg..

[10] Duyan M., Ozturan I.U., Altas M. (2021). Delayed Parosmia Following SARS-CoV-2 Infection: A Rare Late Complication of COVID-19. SN Compr. Clin. Med..

[11] Islek A., Balci M.K. (2021). Phantosmia with COVID-19 Related Olfactory Dysfunction: Report of Nine Case. Indian J. Otolaryngol. Head Neck Surg..

[12] Ercoli T., Masala C., Pinna I., Orofino G., Solla P., Rocchi L., Defazio G. (2021). Qualitative smell/taste disorders as sequelae of acute COVID-19. Neurol. Sci..

[13] Hopkins C., Surda P., Vaira L., Lechien J., Safarian M., Saussez S., Kumar N. (2020). Six month follow-up of self-reported loss of smell during the COVID-19 pandemic. Rhinology.

[14] Raad N., Ghorbani J., Naeini A.S., Tajik N., Karimi-Galougahi M. (2021). Parosmia in patients with COVID-19 and olfactory dysfunction. Int. Forum Allergy Rhinol..

[15] Blomqvist E.H., Brämerson A., Stjärne P., Nordin S. (2004). Consequences of olfactory loss and adopted coping strategies. Rhinology.

[16] Croy I., Hummel T. (2017). Olfaction as a marker for depression. J. Neurol..

[17] Frasnelli J., Hummel T. (2004). Olfactory dysfunction and daily life. Eur. Arch. Otorhinolaryngol..

[18] Qian C., Zhou M., Cheng F., Lin X., Gong Y., Xie X., Li P., Li Z., Zhang P., Liu Z. (2020). Development and multicenter performance evaluation of fully automated SARS-CoV-2 IgM and IgG immunoassays. Clin. Chem. Lab. Med..

[19] Wagner K.I., Mateyka L.M., Jarosch S., Grass V., Weber S., Schober K., Hammel M., Burrell T., Kalali B., Poppert H. (2021). Recruitment of highly functional SARS-CoV-2-specific CD8+ T cell receptors mediating cytotoxicity of virus-infected target cells in non-severe COVID-19. medRxiv.

[20] Walliczek U., Negoias S., Hähner A., Hummel T. (2016). Assessment of Chemosensory Function Using “Sniffin’ Sticks”, Taste Strips, Taste Sprays, and Retronasal Olfactory Tests. Curr. Pharm. Des..

[21] Stuck B.A., Beule A., Damm M., Gudziol H., Hüttenbrink K.-B., Landis B.N., Renner B., Sommer J.U., Uecker F.C., Vent J. (2014). Position paper “Chemosensory testing for expert opinion in smell disorders”. Laryngorhinootologie.

[22] Wong D.K.C., Gendeh H.S., Thong H.K., Lum S.G., Gendeh B.S., Saim A., Husain S. (2020). A review of smell and taste dysfunction in COVID-19 patients. Med. J. Malays..

[23] Tong J.Y., Wong A., Zhu D., Fastenberg J.H., Tham T. (2020). The Prevalence of Olfactory and Gustatory Dysfunction in COVID-19 Patients: A Systematic Review and Meta-analysis. Otolaryngol. Head Neck Surg..

[24] Samaranayake L.P., Fakhruddin K.S., Panduwawala C. (2020). Sudden onset, acute loss of taste and smell in coronavirus disease 2019 (COVID-19): A systematic review. Acta Odontol. Scand..

[25] Agyeman A.A., Chin K.L., Landersdorfer C.B., Liew D., Ofori-Asenso R. (2020). Smell and Taste Dysfunction in Patients with COVID-19: A Systematic Review and Meta-analysis. Mayo Clin. Proc..

[26] Boscolo-Rizzo P., Guida F., Polesel J., Marcuzzo A.V., Antonucci P., Capriotti V., Sacchet E., Cragnolini F., D’Alessandro A., Zanelli E. (2021). Self-reported smell and taste recovery in coronavirus disease 2019 patients: A one-year prospective study. Eur. Arch. Otorhinolaryngol..

[27] Boscolo-Rizzo P., Menegaldo A., Fabbris C., Spinato G., Borsetto D., Vaira L.A., Calvanese L., Pettorelli A., Sonego M., Frezza D. (2021). Six-Month Psychophysical Evaluation of Olfactory Dysfunction in Patients with COVID-19. Chem. Senses.

[28] Rebholz H., Pfaffeneder-Mantai F., Knoll W., Hassel A., Frank W., Kleber C. (2021). Olfactory dysfunction in SARS-CoV-2 infection: Focus on odorant specificity and chronic persistence. Am. J. Otolaryngol..

[29] Parma V., Ohla K., Veldhuizen M.G., Niv M.Y., Kelly C.E., Bakke A.J., Cooper K.W., Bouysset C., Pirastu N., Dibattista M. (2020). More Than Smell—COVID-19 Is Associated with Severe Impairment of Smell, Taste, and Chemesthesis. Chem. Senses.

[30] Reden J., Maroldt H., Fritz A., Zahnert T., Hummel T. (2006). A study on the prognostic significance of qualitative olfactory dysfunction. Eur. Arch. Otorhinolaryngol..

[31] Hong S.-C., Holbrook E.H., Leopold D.A., Hummel T. (2012). Distorted olfactory perception: A systematic review. Acta Oto-Laryngol..

[32] Bonfils P., Avan P., Faulcon P., Malinvaud D. (2005). Distorted Odorant Perception. Arch. Otolaryngol. Head Neck Surg..

[33] Bagnasco D., Passalacqua G., Braido F., Tagliabue E., Cosini F., Filauro M., Ioppi A., Carobbio A., Mocellin D., Riccio A.M. (2021). Quick Olfactory Sniffin’ Sticks Test (Q-Sticks) for the detection of smell disorders in COVID-19 patients. World Allergy Organ. J..

[34] Murphy C., Schubert C.R., Cruickshanks K.J., Klein B.E.K., Klein R., Nondahl D.M. (2002). Prevalence of Olfactory Impairment in Older Adults. JAMA.

[35] Lee M.-H., Perl D.P., Nair G., Li W., Maric D., Murray H., Dodd S.J., Koretsky A.P., Watts J.A., Cheung V. (2021). Microvascular Injury in the Brains of Patients with COVID-19. N. Engl. J. Med..

[36] Brann D.H., Tsukahara T., Weinreb C., Lipovsek M., Berge K.V.D., Gong B., Chance R., Macaulay I.C., Chou H.-J., Fletcher R.B. (2020). Non-neuronal expression of SARS-CoV-2 entry genes in the olfactory system suggests mechanisms underlying COVID-19-associated anosmia. Sci. Adv..

[37] Yousefi-Koma A., Haseli S., Bakhshayeshkaram M., Raad N., Karimi-Galougahi M. (2021). Multimodality Imaging With PET/CT and MRI Reveals Hypometabolism in Tertiary Olfactory Cortex in Parosmia of COVID-19. Acad. Radiol..

[38] Meinhardt J., Radke J., Dittmayer C., Franz J., Thomas C., Mothes R., Laue M., Schneider J., Brünink S., Greuel S. (2021). Olfactory transmucosal SARS-CoV-2 invasion as a port of central nervous system entry in individuals with COVID-19. Nat. Neurosci..

[39] Butowt R., Meunier N., Bryche B., von Bartheld C.S. (2021). The olfactory nerve is not a likely route to brain infection in COVID-19: A critical review of data from humans and animal models. Acta Neuropathol..

[40] Dehgani-Mobaraki P., Zaidi A.K., Yadav N., Floridi A., Floridi E. (2021). Longitudinal observation of antibody responses for 14 months after SARS-CoV-2 infection. Clin. Immunol..

[41] Miwa T., Furukawa M., Tsukatani T., Costanzo R., Dinardo L.J., Reiter E.R. (2001). Impact of Olfactory Impairment on Quality of Life and Disability. Arch. Otolaryngol. Head Neck Surg..

[42] Altundag A., Cayonu M., Kayabasoglu G., Salihoglu M., Tekeli H., Saglam O., Hummel T. (2015). Modified olfactory training in patients with postinfectious olfactory loss. Laryngoscope.

[43] Hummel T., Rissom K., Reden J., Hähner A., Weidenbecher M., Hüttenbrink K.-B. (2009). Effects of olfactory training in patients with olfactory loss. Laryngoscope.

[44] Damm M., Pikart L.K., Reimann H., Burkert S., Önder G., Haxel B., Frey S., Charalampakis I., Beule A., Renner B. (2013). Olfactory training is helpful in postinfectious olfactory loss: A randomized, controlled, multicenter study. Laryngoscope.

[45] Geißler K., Reimann H., Gudziol H., Bitter T., Guntinas-Lichius O. (2013). Olfactory training for patients with olfactory loss after upper respiratory tract infections. Eur. Arch. Otorhinolaryngol..

[46] Xydakis M.S., Albers M.W., Holbrook E.H., Lyon D.M., Shih R.Y., Frasnelli J.A., Pagenstecher A., Kupke A., Enquist L.W., Perlman S. (2021). Post-viral effects of COVID-19 in the olfactory system and their implications. Lancet Neurol..

